# Fulminant Clostridium Septicum myonecrosis in well controlled diabetes: a case report

**DOI:** 10.1186/1752-1947-1-119

**Published:** 2007-10-30

**Authors:** Huy A Tran, Esther Myint

**Affiliations:** 1 Division of Clinical Chemistry Hunter Area Pathology Service, John Hunter Hospital, Locked Bag 1, Hunter Region Mail Centre, Newcastle, New South Wales, Australia 2310; 2 Division of Anatomical Pathology Hunter Area Pathology Service, John Hunter Hospital, Locked Bag 1, Hunter Region Mail Centre, Newcastle, New South Wales, Australia 2310

## Abstract

Diabetic myonecrosis with Clostridium Septicum is uncommon but carries a high mortality rate. This commensal organism is part of the gastrointestinal tract flora and can become extremely virulent, often in the setting of immuno-suppression such as neutropenia, occult malignancy (commonly caecal) and poorly controlled diabetes. The case report is unusual in that there are few risk factors other than very mild neutropenia. This highlights the opportunistic character of the organism and recommends that a high index of suspicion and vigilance be carried out in the presence of fevers and sepsis, even in the well-controlled diabetic population.

## Background

The case reports a unique and peculiar condition of extensive and fatal Clostridium Septicum myonecrosis in a diabetic patient undergoing chemotherapy for her breast cancer. Contrary to previous reports where the common risk factors are poorly controlled diabetes, severe neutropenia and classically caecal carcinoma, a sanctuary for the bacillus, our patient had only mild and asymptomatic neutropenia. Another notable risk factor was morbid obesity in the patient which confers a general increase in all-risk mortality. Hyperinsulinaemia in the setting of insulin resistance and obesity is another critical factor, thought to differentially inhibit neutrophil function. Whilst each factor is modest on its own, the combination delivers a fatal outcome in this case once the organism has disseminated.

## Case presentation

A 68 year-old woman with type 2 diabetes mellitus and intraductal breast carcinoma presented with a 2-day history of rapidly onset left groin pain and leg weakness. There were some discoloration and blisters on the medial aspect of the same thigh and she had felt warm but denied any rigors. She became unable to weight bear and began to vomit on the day of presentation. No history of trauma was noted to the area. Her past medical history included type 2 diabetes of eight years with reasonable control, requiring Metformin 1 gm twice daily. Her home daily glucose readings oscillated between ~5–7 mmol/L in the previous 6–8 weeks, with a HbAc1 of 6.5% two weeks prior. Serial levels had been between 6.2 and 7.1% in the previous 2 years. The only diabetes complication included mild background non-proliferative retinopathy which has been observed. Her breast cancer was diagnosed 6 months prior with grade 3 intraductal carcinoma with axillary metastasis requiring mastectomy with axillary clearance followed by systemic adjuvant chemotherapy with Adriamycin and Cyclophosphamide. Her last course of chemotherapy was 10 days prior with no mucositis or any other adverse effects. Past medical problems included hypertension and diverticular disease requiring partial sigmoid bowel resection 6 years ago.

**Figure 1:**
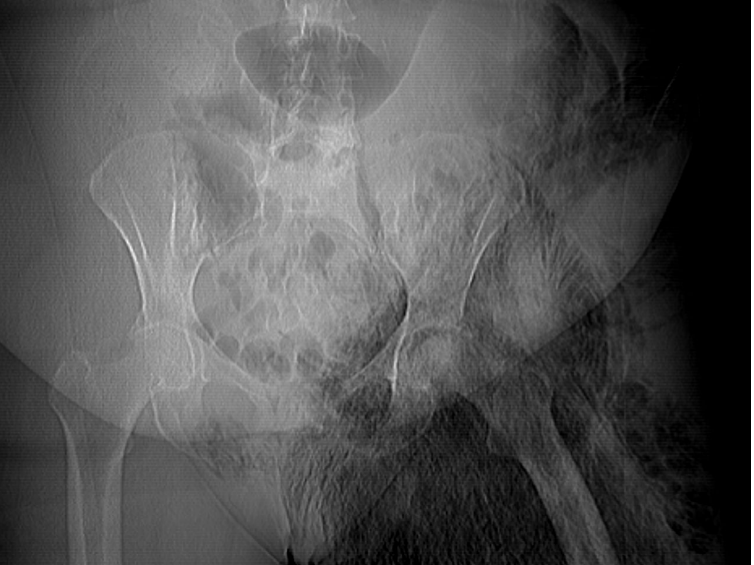
Plain XR showing extensive and ominous gaseous formation in the left pelvis and thigh.

**Figure 2:**
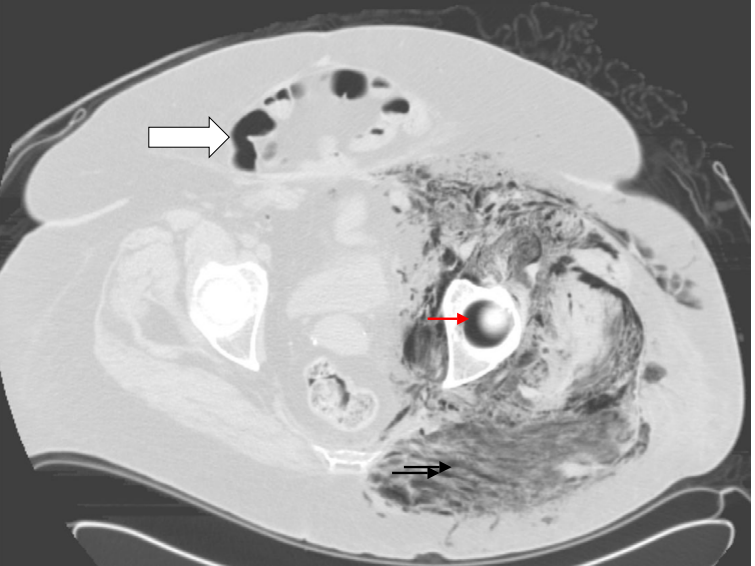
CT scan of the pelvis confirming the extensive gaseous formation tracking along the fascial planes characteristic of myonecrosis *(black arrows)*. The formed gas also extends into the acetabular gap, giving the crescentic appearance *(red arrow)*. Note the marked adiposity in the form of anterior fat-apron with a ventral wall hernia *(large arrow)*.

Examination showed an ill-looking and obese woman with pyrexia of 38.8° Celsius, pulse rate of 110 beats per minute, regular and blood pressure of 100/70 supine. No standing blood pressure was possible. Her body mass index (BMI) was ~36 kg/m^2 ^(weight 98.1 kg with height 1.64 m). The lower limb was markedly swollen with extensive ecchymosis extending from the left groin inferiorly to the mid thigh. The dorsalis pedis and posterior tibial arterial pulses were present. Crepitus was detected on the medial aspect of her thigh. Investigations showed a haemoglobin of 94 g/L (reference range [RR], 115–165), white cell count 4.0 × 10^9^/L (RR, 4.0 – 11.0), neutrophils 3.4 × 10^9^/L (RR, 4.0–7.0 × 10^9^/L), platelet 197 × 10^9^/L (RR, 150–450). Her sodium was 134 mmol/L (RR, 137 – 143), potassium 3.8 mmol/L (RR, 3.5 – 5.5), urea 8.1 mmol/L (RR, 2.6 – 6.4) and creatinine 142 umol/L (RR, 60 – 100), C-reactive protein 235 ug/L (RR, < 3), Creatine Kinase 1668 U/L (RR, < 125) and a random glucose of 22.4 mmol/L. Pelvic XR revealed diffuse gas formation extending inferiorly down the left leg (Figure 1), further supported with computerized tomographic scans (Figure 2).

**Figure 3:**
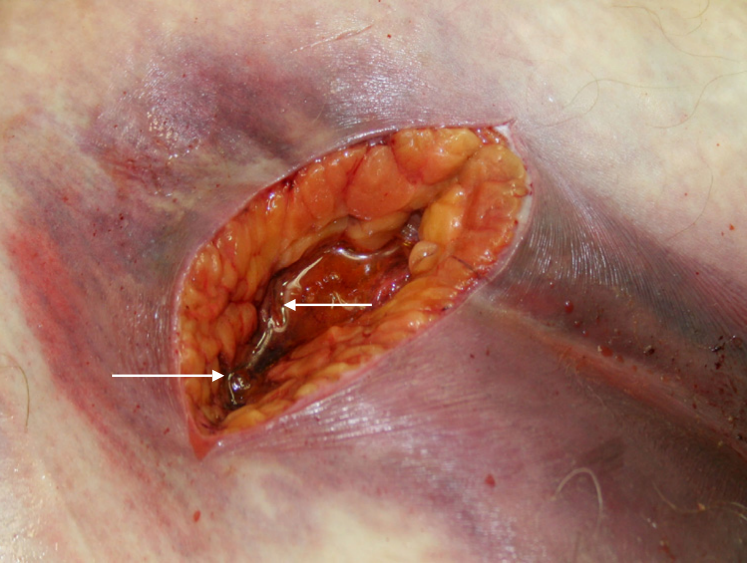
Post mortem incision of the necrotic tissue exudes an abundance of gaseous bubbles *(arrows)*.

Intravenous antibiotics including Penicillin, Gentamicin and Metronidazole were urgently administered together with crystalloid rehydration. Surgery was seriously considered but withheld due to the extensive nature of the infection. Not unexpectedly, the patient died of overwhelming sepsis in 12 hours despite rigorous and intensive medical therapy. Hyperbaric therapy was not available. Blood culture subsequently returned positive for Clostridium Septicum. Post mortem examination revealed extensive myofascial necrosis and subcutaneous gaseous formation (Figure 3). Staining study revealed an abundance of gram-positive bacilli in the necrotic tissue supporting the blood culture finding. No bowel malignancy was detected.

## Discussion

Necrotising fasciitis is a critical condition involving the Clostridial species. Cl. Septicum is part of the normal gastrointestinal tract flora but when infection occurs, it is often fatal, especially in the diabetic population. The infection is well documented in other immunocompromised states such as leukaemia, post-chemotherapy neutropenia and occult malignancies, often colonic [1,2]. Contrary to its close cousin, Cl. Perfringens which is strictly anaerobic, Cl. Septicum is relatively more aerotolerant and thus can survive much longer as spores in normal and consequently can infect healthy tissues. It is also more virulent because the inoculum required for Cl. Septicum to cause an infection is ~300 times lower in mice compared with Cl. Perfringens [3]. It flourishes in tumor tissue because there is often marked anaerobic glycolysis occurring. Focal area of necrosis present in such tissue may also be hypoxic, accelerating the spread of the organism. The presence of poorly controlled diabetes can aggravate the risk by the poor vascular supply with relative ischaemia of the muscle. Where there is persistent hyperglycaemia, leucocytes do not function optimally, further aggravating the risk of systemic sepsis. In neutropenic sepsis and haematologic malignancies, there are often abrasions and ulcerations of bowel mucosa allowing the organism a port of entry into the circulation. This species is made more virulent by producing large amount of toxins and proteolytic enzymes, further accelerating its spread [4]. The DNases β-toxin is thought to assist in spreading through the tissue planes and hyaluronidase δ-toxin in the muscular tissue destruction respectively.

Another important factor is the patient's morbid obesity, which contributes to her both primary diagnoses of breast cancer and well controlled type 2 diabetes [5]. Although not directly responsible for the fasciitis, the obesity also markedly amplifies her risk of all cause mortality [6]. Furthermore, obese patients appear to under-report their own health status, even in the absence of chronic disease, which may have delayed the patient's presentation with a subsequent fatal outcome [7].

The case is unusual in two aspects. The first is the complete absence of trauma or the presence of a colonic malignancy. Secondly, her diabetes control had been satisfactory as reflected by the biochemical parameters. This is thought to be due to the differential effect of hyperinsulinaemia in the setting of insulin resistance on neutrophil function, irrespectively of glycaemic status [8,9]. Thus, it is postulated that in the presence of mild neutropenia, whose function is inhibited by effect of insulin excess, the bacillus was able to find a port of entry, probably via micro-abrasions of the bowel mucosal lining. It then is able to systemically and overwhelmingly disseminate by the mechanisms previously described.

Because of this accelerated spread of infection, most cases invariably succumb to overwhelming sepsis with a mortality exceeding 80%. Outcome therefore critically depends upon early recognition and diagnosis of the condition. Treatments should include aggressive resuscitation, prompt institution of the appropriate combination antibiotics and radical surgical debridement. Hyperbaric therapy should also be considered where available. In paediatric patients where there are often fewer co-morbidities, the situation can sometime be salvaged with extensive surgery [10].

## Conclusion

The case highlights the grave prognosis of Clostridial sepsis, especially where there are associated co-morbidities. It is critically important that this highly fatal condition is considered early and frequently in septic patients, even in the presence of well controlled diabetes so that early and appropriate therapy can be implemented. With the increasing immunocompromised population, compounded by the exploding epidemic of type 2 diabetes, obesity and breast tumor being the commonest cancer in females, the incidence of this peculiar infection can only be expected to escalate.

## Competing interests

The author(s) declare that they have no competing interests.

## Authors' contributions

EM gathered, provided the data and participated in the discussion and drafting of the manuscript. HAT conceived and designed the case report, assisted with data collection, coordinated and helped to draft the manuscript. Both authors read and approved the revised manuscript.

## Consent

Written informed consent was obtained from the patient's next of kin.
